# A long-lasting gel-based diffuser of feline pheromone can help reduce undesirable behaviors in cats at home: comparison with an electric diffuser

**DOI:** 10.3389/fvets.2024.1445108

**Published:** 2024-08-29

**Authors:** Gemma Espuña, Céline S. Nicolas, Aurélie Girardin, Jaume Fatjó, Jonathan Bowen, Patricia Monginoux, Christophe A. Rème

**Affiliations:** ^1^GM&MD Department, Virbac SA, Carros, France; ^2^MU Petfood Petcare, Virbac SA, Carros, France; ^3^Ethometrix Ltd., East Sussex, United Kingdom

**Keywords:** electric-free diffuser, Zenifel, feline facial pheromone, relaxing effects, situational stress, easiness of use, catnip, long-term use

## Abstract

Using pheromone diffusers is part of the strategy to control stress-related behaviors in cats (*Felis catus*). The goal of the study was to compare the efficacy of a novel long-acting, unpowered gel-based diffuser containing a facial pheromone analog (Zenifel^®^ gel diffuser, Virbac) with a similar electrically powered feline pheromone diffuser that already has proven efficacy, on situational stress in cats, at home. The study involved 90 owners of cats presenting undesirable behaviors receiving one diffuser or the other: 46 received one gel diffuser and 44 received one plug-in device and a refill, for the 2-month study duration. The presence of the various undesirable behaviors was checked regularly and a general score was given to rate their presence. The most reported behaviors were related to hypervigilance, seeking the owner’s attention, hiding, excessive vocalization, inactivity, and excessive scratching. All six behaviors significantly improved over time with Zenifel^®^ while only four improved with the reference product (no improvement for excessive meowing and inactivity). The general score significantly improved as of day 7 in both groups, with no difference between groups. More owners said they would use the product throughout the year with Zenifel^®^ than with the reference product (80% vs. 42%, *p* < 0.05). Therefore, both diffusers can be used to help control undesirable behaviors of cats at home but Zenifel^®^ is more convenient to use throughout the year.

## 1 Introduction

There are few reliable statistics on the overall prevalence of behavior problems in cats (*Felis catus*), but in a large scale epidemiological study using data from primary-care veterinary clinics in the UK, 2.7% of cats were recorded as having undesirable behavior (1). In that survey, another 4.4% of cats had lower urinary tract disorders and 4.7% had upper respiratory tract disorders, both of which can have an underlying stress component. Abscesses were recorded in 6.5% of cats, and those not only mostly result from inter-cat conflict, but also reflect the end result of stressful interactions between cats that can contribute to other problems such as indoor spray-marking. However, as reported by Grigg et al. (2), very few owners seek behavioral help for their cats (<4%), while the majority (98%) of the 448 owners replying to the survey reported that their cats had at least one behavioral problem. The prevalence of behavioral problems is therefore high and underestimated when assessing only veterinarians.

Stress can impact the cat’s quality of life and can be a risk factor for a number of diseases. For example, stress plays an important role in the reactivation of the feline herpes virus, a frequent cause of respiratory disease (3). Stressed cats are more than five times more likely to develop an upper respiratory tract infection than cats with lower levels of stress (4). Stress has also been linked to a number of medical conditions, including feline idiopathic ulcerative dermatitis, gastrointestinal problems (e.g., diarrhea and vomiting), dermatological diseases (e.g., atopic dermatitis and acral dermatitis) and feline idiopathic cystitis (FIC), the most common diagnosis in cats with feline lower urinary tract disease (5–7).

Behavioral problems can have serious consequences for owned cats. For example, in a large survey in the UK, house-soiling, including inappropriate urine marking, was found to be the most common behavioral problem in cats, as well as the most common behavioral reason for both the rehoming of cats and their return to the shelter after unsuccessful adoption (8). Taken together, these findings indicate that stress is of significance to the well-being of domestic cats. The primary methods of treating behavioral problems in cats are environmental modification and enrichment (6, 9), but there is a need for effective biological therapies to support these approaches, primarily by reducing anxiety and stress. Biological therapies or management can include prescription psychoactive medication, supplements (including herbal supplements) and pheromones (10).

Pheromones are individual molecules or a set of chemical compounds that are excreted on the outside of an individual’s body, are received by a member of the same species and activate a specific behavioral response in that receiving conspecific (11). These responses often relate to reproduction, territory marking, or social interactions. Pheromones are detected by the vomeronasal organ (12) and play an important role in behavior regulation by modifying perception, emotion, and motivation (11). Pheromone analogs can be interesting tools to soothe pets as they are safe to use and have some relative efficacy. Since the 1990s, synthetic analogs of feline facial pheromones have been produced and marketed mainly in the form of diffusers or sprays with soothing and relaxing claims (13). Some studies have shown the effectiveness of the F3 feline facial pheromone analogs in reducing a range of undesirable behaviors in cats, including destructive scratching (14), urine marking (15), and situational stress (16, 17). Due to their convenience and safety profile, pheromones could therefore be considered to be first-line substances for the behavioral management of cats showing either mild signs of stress and behavioral problems, or for stress prevention. Depending on the case, pheromones can be used alone or in combination with nutraceuticals or prescription medicines (6).

Owner acceptance of treatments is critical to adherence and a successful outcome. In a study of the perceptions and attitudes of owners toward different biological therapies, 74% of respondents were comfortable with the use of pheromone products, compared with 50% being comfortable with long-term prescription medication and 52% being comfortable with cannabinoid products (2). The greatest concerns respondents had about the use of prescription medication were about side effects and sedation, but the decision to give medication was also strongly influenced by the difficulty of administration (2). Pheromone products are not administered to the animal directly, so they do not present the same challenges as other biological therapies. Pheromone diffusers typically last for 1 month, but chronic stress can take longer than this to manage. An effective behavior management program would therefore require several refills or replacements of these short-term devices. Alternatives have now been developed to provide a longer-lasting effect in a more environmentally friendly presentation. Zenifel^®^ gel diffuser (Virbac^®^) lasts for 2 months and requires no electricity to operate.

In order to test the efficacy of the new Zenifel^®^ product and compare it to a plug-in product with proven efficacy [Feliway^®^ Optimum (18)], we conducted a 2-month study in 90 owned cats exhibiting “signs of non-specific anxiety/distress” as identified in the 2015 AAHA Canine and Feline Behavior Management Guidelines (10). One group received the electric pheromone diffuser and a refill while the second group received the 2-month Zenifel^®^ Gel Diffuser (Virbac^®^; France). Undesirable behaviors were assessed regularly by the owners using questionnaires.

## 2 Materials and methods

### 2.1 Animals and selection

The study was conducted in France by Techni’Sens (La Rochelle, France). Participants were recruited by Techni’Sens from the company’s proprietary panel of 50,000 households using the following screening criteria. Only non-smoking owners with a single, non-aggressive cat (and with no dog living in the house) were permitted as participants. Recruited cats had to be aged >6 months old, live mainly indoors, and not be currently treated for behavioral problems, but could be of any breed or sex (but not pregnant or lactating). Inclusion was based on the owner’s responses to a pre-study questionnaire that assessed the general behavior of the cats. There were three sets of behavioral criteria for inclusion of a cat:

- The owner reported that their cat presented with at least one of the following five characteristics: fearful, anxious, stressed, suspicious, or destructive (e.g., scratching furniture).
- The cat also had to present with at least one behavior from a list of 25 behaviors that have been previously described as commonly recognized signs of nonspecific anxiety/distress (Table 1) (10).
- The owners gave a score of 5 or above on a scale from 0 to 10 when asked to score the overall presence of those signs, with 0 being “Not present” and 10 being “Very present.”

**Table 1:** List of behaviors that have been previously described as commonly recognized signs of nonspecific anxiety/distress [after Hammerle et al. (10)].

List of behaviors for inclusion of cats in the study	
Hypervigilance/hyperalertness, scanning environment	Urinating in an unusual/inappropriate place
Seeking the owner’s attention	Defecating in an unusual/inappropriate place
Hiding or trying to hide	Decreased contact with the owner
Repetitive or excessive meowing out of context	Often licks lips and nose
Inactivity (motionless, with no activity and not playing)	Smacks/pops lips or jaws together (chatters teeth)
Excessive scratching	Does not tolerate direct eye contact
Raises hair and twitches skin	Retracts lips/grimaces
Carries the head and/or the neck low, tail between the legs	Repetitive activities (turn in circle, tail-chasing, pacing)
Tries to escape	Panting (even if not hot weather)
Hyperactivity	Often discharges anal glands (glands located near the anus with a bad odor)
Overgrooming	Trembles a lot
Decreased grooming	Drools excessively
Mydriasis (dilated pupils)	

One hundred owners of healthy cats were initially recruited and 90 completed the 2-month study.

### 2.2 Products and groups

One group of 46 owners received one non-electric diffuser (Zenifel^®^ Gel Diffuser, Virbac^®^, containing 6% F3 fraction of feline facial pheromone analog and 0.5% of *Nepeta Cataria* extract, with a 2-month duration) and the other group of 44 owners received an electric pheromone diffuser (Feliway Optimum^®^; Ceva^®^, containing 2% feline pheromone analog complex, with a 30-day duration) and one refill (for a total duration of 2 months) as a reference product. Both products were unbranded (no label on them, except for the inscribed names on the diffuser—Feliway—or lid—Zenifel). Owners knew they were testing a product designed to appease the cat but did not know the composition of the products. They were instructed to place the supplied diffuser in the room where the cat spends most of its time.

### 2.3 Procedures

On Days 0, 7, 15, 30, 45, and 60, the owners completed questionnaires to assess the behavior of their cat. Each time, the owners had to select which of the behaviors listed previously (see animals and selection) were still present. Then, they had to give a score (from 0 to 10) to rate the presence of the signs. The owners could also select any new behavior(s) and provide a score for them.

At the end of the study, owners could say if the cat seemed calmer in some specific situations. They could reply “yes,” “no” or “not applicable.” The percentage of owners replying “yes” was calculated, excluding those replying “not applicable.” The owners could also select some situations in which they would use the diffuser in the future. Other questions related to the product’s characteristics, like the odor or reaction of the cat toward the diffuser, and easiness of use, were also asked at the end of the study.

### 2.4 Statistical analysis

Categorical data were compared between groups using Fisher’s exact test or chi-square tests.

The number of signs reported in the two groups on Day 0 was compared with a Mann–Whitney test.

Presence of behaviors over time: the distribution of owners reporting a specific behavior (listed in 2.1 Animals and selection) at each time point of assessment was analyzed using a Cochran’s Q test. This test compares the response to two treatments, to determine whether the proportion of successful outcomes is the same. Mc Nemar’s tests were then used to compare each time point of assessment with day 0. Only the signs reported by at least 8 owners per group on Day 0 were analyzed.

Scores: At each time point of assessment, the scores given for the presence of undesirable behaviors previously reported and for new ones were multiplied by the number of signs reported (previously reported and new ones, respectively). The scores for previous and new signs were then added and divided by the number of signs ever reported (on day 0 and later) to get an adjusted global score. Due to the ordinal nature and non-normal distribution of the data (verified with the Shapiro–Wilk test), only non-parametric tests were used. Friedman tests, followed by Wilcoxon signed-ranks tests in case of significance, were used to assess the difference in adjusted global scores with day 0 in each group (intra-group comparison). Groups were compared at each time point using Mann–Whitney tests on the percentage of improvement of the adjusted global score from day 0.

The threshold for statistical significance was set for *p* < 0.05. When necessary, a correction for 5 comparisons (time points of assessment vs. day 0) was applied to assess statistical significance, using Benjamini-Hochberg’s adjustment method.

Data are presented as percentages or median (first quartile Q1; third quartile Q3).

The data analysis for this paper was generated using the Real Statistics Resource Pack software (Release 8.9.1). Copyright (2013–2024) Charles Zaiontz.^1^

## 3 Results

### 3.1 Characteristics of selected cats

The characteristics of the recruited cats per group are depicted in Table 2. Overall, there were more females (60%), spayed cats (96%), young or medium aged (<9 years old, 74%), and cats weighing less than 5 kg (57%). Most cats were described as fearful (67%) and/or suspicious (50%) and fewer cats were described as stressed (39%), anxious (28%) or destructive (e.g., scratching furniture) (20%). Sixty percent of cats were also described as calm, 59% as cuddly, and 38% as playful. The median (Q1; Q3) number of undesirable behaviors reported by all owners was 3 (2;4). There was no significant difference between groups at baseline for any of the criteria (*p* > 0.05 for all; Table 2).

**Table 2:** Characteristics of cats recruited on day 0.

	Zenifel gel diffuser	Reference electric diffuser
Total number of cats	46	44
Sex		
Male	18 (39%)	18 (41%)
Female	28 (61%)	26 (59%)
Neutered/spayed		
Yes	44 (96%)	42 (95%)
No	2 (4%)	2 (5%)
Age		
≤ 5 years old	23 (50%)	18 (41%)
6 to 9 years old	11 (24%)	14 (32%)
10 to 14 years old	10 (22%)	10 (22%)
≥ 15 years old	2 (4%)	2 (5%)
Body weight		
< 5 kg	25 (54%)	26 (59%)
> 5 kg	21 (46%)	18 (41%)
Cat temperament		
Fearful	27 (59%)	33 (75%)
Suspicious	20 (43%)	25 (57%)
Stressed	16 (35%)	19 (43%)
Anxious	11 (24%)	14 (32%)
Destructive (scratching, etc.)	8 (17%)	10 (23%)

Data are presented as the number of cats (%) in each group. There was no significant difference between groups for any of the characteristics (*p* > 0.05).

The undesirable behaviors reported by owners on day 0 per group are reported in Table 3 and Supplementary Table 1. Overall, the most cited behaviors were: hypervigilance/hyperalertness (60%); seeking owner’s attention (44%); hiding or trying to hide (34%); repetitive or excessive meowing (29%); inactivity (23%); and excessive scratching (19%). There was no statistical difference between the two groups with respect to the percentage of owners reporting these six individual behaviors (*p* > 0.05 for all behaviors; Table 3) or between the number of undesirable behaviors reported per group when considering only these six main behaviors (with a median of 2 in each group, *p* = 0.9). Other behaviors were reported by less than 15% of owners (see Supplementary Table 1) and were not analyzed further. The situations where the undesirable behaviors occurred are depicted in Supplementary Table 2 (mainly when there is a loud noise or with unfamiliar people).

**Table 3:** Most cited undesirable behaviors.

Behavior	Zenifel gel diffuser (*n* = 46)	Reference electric diffuser (*n* = 44)
Hypervigilance/hyperalertness	25 (54%)	29 (66%)
Seeking owner’s attention	19 (41%)	21 (48%)
Hiding or trying to hide	16 (35%)	15 (34%)
Repetitive or excessive meowing out of context	14 (30%)	12 (27%)
Inactivity	13 (28%)	8 (18%)
Excessive scratching	9 (20%)	8 (18%)

Only the behaviors that have been reported by more than 15% of owners are reported here. Data are presented as the number of cats (%) in each group. There was no significant difference between groups for any of the behaviors (*p* > 0.05).

The general score given for the undesirable behaviors on day 0 was of 6 (6; 8) in the Zenifel group and of 7 (6; 8) in the reference group (no significant difference between groups, *p* = 0.4).

### 3.2 Evolution of behaviors and scores

**Figure 1:**
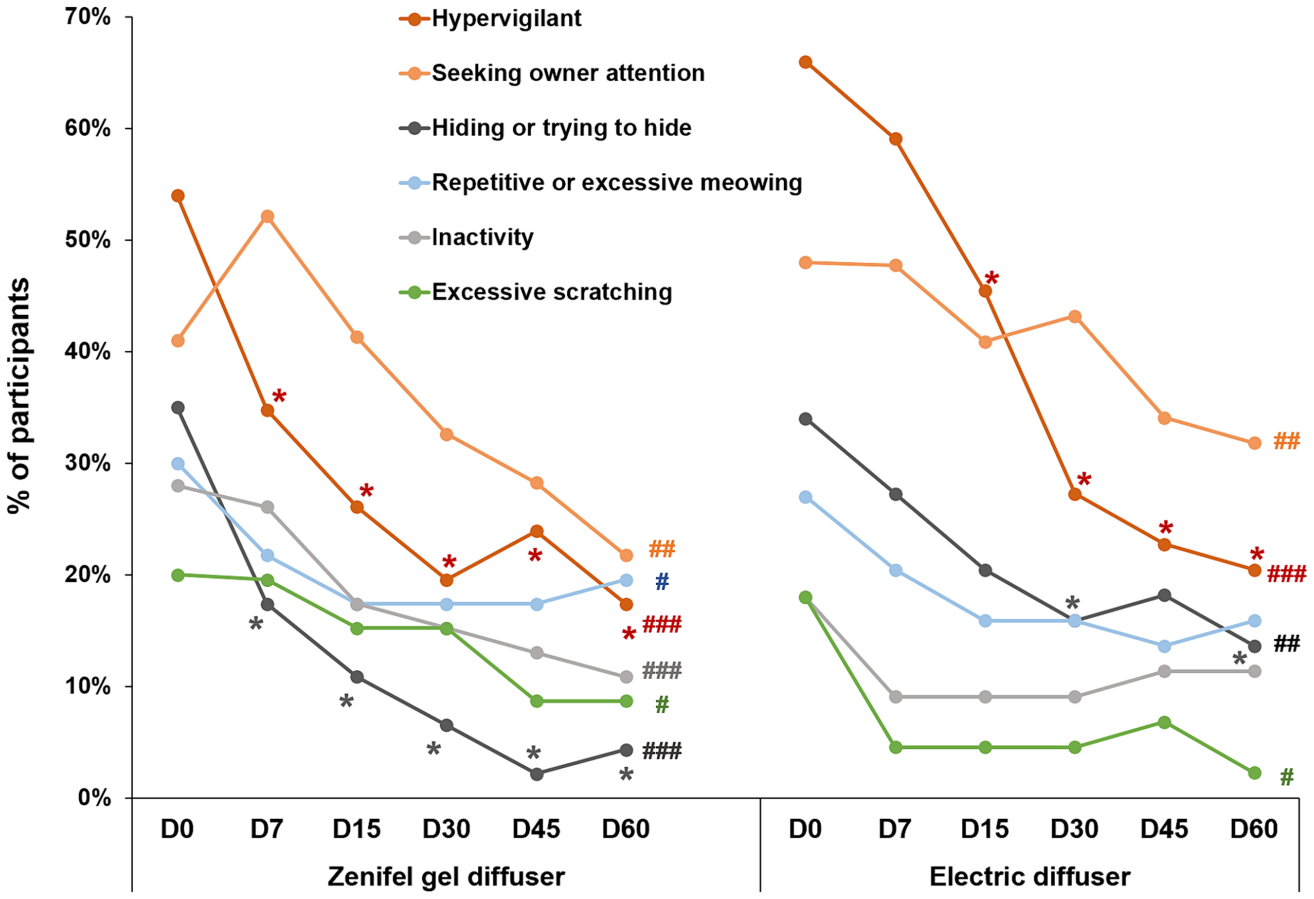
Percentage of owners reporting the most cited behaviors on each assessment day. #, ##, ### Significant change in proportion over time (#*p* < 0.05; ##*p* < 0.01; ###*p* < 0.001). * Significant difference versus day 0 (D0) after adjusting for multiple comparisons (*p* < 0.05).

The proportion of owners reporting the six most cited undesirable behaviors significantly decreased over time in the Zenifel group (*p*-values ranging from <0.0001 to 0.03, Cochran *Q* test, Figure 1). The number (%) of owners reporting each behavior is reported below (see also Figure 1):

- “hypervigilance/hyperalertness” went from 25 (54%) to 8 (17%) between day 0 and day 60 (overall significant decrease: *p* < 0.0001, Cochran Q test). Comparing each data independently versus day 0 showed that this decrease was significant as of day 7 (*p* = 0.016, Mc Nemar’s test), even after adjusting for multiple testing.
- “Seeking owner’s attention” went from 19 (41%) to 10 (22%) (*p* < 0.0001). The greatest decrease was observed on day 60 (*p* = 0.027—not significant (NS) after adjusting for multiple testing).
- “Hiding or trying to hide” went from 16 (35%) to 2 (4%) between Day 0 and Day 60 (*p* < 0.0001). This decrease was significant as of day 7 (*p* = 0.013), even after adjusting for multiple testing.
- “Repetitive or excessive meowing” went from 14 (30%) to 9 (20%) (*p* = 0.03). The greatest improvement was observed on days 15–45 [8 (17%) – *p* = 0.077, NS].
- “Inactivity” went from 13 (28%) to 5 (11%) (*p* < 0.0001). The greatest decrease was observed on day 60 (*p* = 0.077, NS).
- “Excessive scratching” went from 9 (20%) to 4 (9%) (*p* = 0.019). The greatest decrease was observed on days 45 and 60 [4 (9%), *p* = 0.13, NS].

In the reference group, 4 out of the 6 most cited behaviors significantly decreased over time, according to the proportion of owners reporting them (see also Figure 1):

- “Hypervigilance/hyperalertness” went from 29 (66%) to 9 (20%) between Day 0 and Day 60 (*p* < 0.0001, Cochran Q test). This decrease was significant as of day 15 (*p* = 0.026, Mc Nemar’s test), even after adjusting for multiple testing.
- “Seeking owner’s attention” went from 21 (48%) to 14 (32%) between Day 0 and Day 60 (*p* = 0.015). The greatest decrease was observed on day 60 (*p* = 0.046 – NS after adjusting for multiple testing).
- “Hiding or trying to hide” went from 15 (34%) to 6 (14%) between Day 0 and Day 60 (*p* = 0.0013). Significant decreases were observed on day 30 [7 (16%), *p* = 0.013] and 60 [6 (14%), *p* = 0.008].
- “Excessive scratching” went from 8 (18%) to 1 (2%) between Day 0 and Day 60 (*p* = 0.0024). The greatest decrease was observed on day 60 (*p* = 0.023 – NS after adjusting for multiple testing).
- “Repetitive or excessive meowing” went from 12 (27%) to 7 (16%) (*p* = 0.19, NS). The greatest improvement was observed on day 45 [6 (14%) – *p* = 0.08, NS].
- “Inactivity” went from 8 (18%) to 5 (11%) (*p* = 0.09, NS). The greatest decrease was observed on days 7, 15 and 30 [4 (9%), *p* = 0.13, NS].

There was no difference between groups (*p* > 0.05) when comparing the change in proportion of owners expressing each behavior on day 60. The success rates (number of owners which do not see the behavior on day 60 while they reported it before) in the Zenifel and reference groups were:

- “Hypervigilance/hyperalertness”: 17/25 (68%) vs. 20/29 (69%) in the Zenifel and reference groups, respectively (*p* = 1);
- “Seeking owner’s attention”: 9/19 (47%) vs. 7/21 (33%), respectively (*p* = 0.5);
- “Hiding or trying to hide”: 14/16 (88%) vs. 9/15 (60%), respectively (*p* = 0.1);
- “Excessive scratching”: 5/9 (56%) vs. 7/8 (88%), respectively (*p* = 0.3);
- “Repetitive or excessive meowing”: 5/14 (36%) vs. 5/12 (42%), respectively (*p* = 1);
- “Inactivity”: 8/13 (62%) vs. 3/8 (38%), respectively (*p* = 0.4).

**Figure 2:**
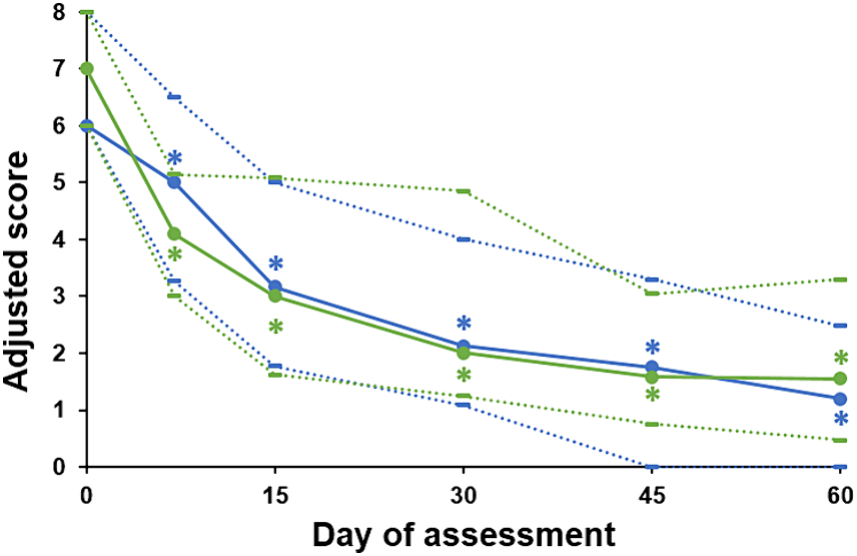
Adjusted global scores obtained on each assessment day for undesirable behaviors. Data are presented as medians (plain lines), first quartile (dotted lines below medians) and third quartile (dotted lines above medians) for the Zenifel^®^ group (blue lines) and reference group (green lines). The scores significantly decreased over time in both groups (*p* < 0.001), with no difference between groups. * Significant difference versus day 0 after adjusting for several comparisons (*p* < 0.05).

The adjusted global scores calculated for the undesirable behaviors also significantly decreased over time in both groups (*p* < 0.0001 for both), with a significant decrease as of day 7 with both diffusers (*p* < 0.0001 in both groups, Figure 2). The median score went from 6 to 1.2 (80% decrease) in the Zenifel^®^ group (*p* < 0.0001) and from 7 to 1.55 in the reference group (78% decrease) from day 0 to day 60 (*p* < 0.0001). There was no difference between groups (*p* > 0.05) when comparing the percentage of evolution of this score from day 0, at any assessment day. The median (Q1-Q3) score change was of: −28% (−49%; 0) vs. −40% (−57%; −19%) in the Zenifel and reference groups, respectively, on day 7 (*p* = 0.1); −51% (−72%; −23%) vs. −59% (−73%; −32%), respectively, on day 15 (*p* = 0.5); −67% (−85%; −34%) vs. −69% (−83%; −36%), respectively on day 30 (*p* = 0.6); −75% (−100%; −45%) vs. −75% (−91%; −56%), respectively on day 45 (*p* = 0.7); and − 85% (−100%; −60%) vs. −75% (−94%; −64%), respectively on day 60 (*p* = 0.6).

### 3.3 Situations of use

At the end of the study, the owners were asked to say whether the cats seemed more appeased in some specific situations. Overall, more than half of owners replied that their cats seemed more appeased when left alone (58%), with unfamiliar people (55%), when touched or carried (55%), during transportation (55%), with other animals (51%) or in another situation (55%). Other situations identified were: during visits to the veterinarian or other people (47%), during a change of environment (47%), when there is a loud noise (40%), when feeling threatened (36%), or when forced to stay in a place or cage (34%). There was no significant difference between groups (*p* > 0.05) for any of these situations (Table 4).

**Table 4:** Situations where the cat seems calmer.

Situation	Zenifel gel diffuser	Reference electric diffuser
When left alone	21/40 (52%)	25/40 (63%)
With unfamiliar people	20/38 (53%)	22/38 (58%)
For no particular reason	18/34 (53%)	20/35 (57%)
When touched or carried	23/45 (51%)	24/41 (59%)
During transportation (car or other)	6/13 (46%)	10/16 (63%)
With other animals	6/13 (46%)	12/22 (55%)
During visit to the veterinarian or other person	8/19 (42%)	8/15 (53%)
Change of environment	11/22 (50%)	9/21 (43%)
Loud noise (firework, thunder, hoover…)	18/43 (42%)	16/41 (39%)
When feeling threatened	12/28 (43%)	9/31 (29%)
When forced to stay in a particular place or cage	5/15 (33%)	10/29 (34%)
Other	7/16 (44%)	6/16 (38%)

Number (%) of owners replying their cat seemed calmer in each specific situation (among owners replying yes or no and excluding those replying “Not applicable”). There was no significant difference between groups for any of the situation (*p* > 0.05).

When asked how they would use the product they tested after purchasing, 57% said they would use it to decrease undesirable behaviors and the same percentage would use it throughout the year. Twenty-six percent would use it when moving in a new home or with another pet, 25% would use it before a long trip and 11% would use it when they had guests visiting.

There was no significant difference between groups (*p* > 0.05) except for the use throughout the year, for which more participants answered positively in the Zenifel group (*p* = 0.01; Table 5).

**Table 5:** Situations when the owners would use the product.

Situation	Zenifel gel diffuser (*n* = 20)	Reference electric diffuser (*n* = 33)
To decrease undesirable behaviors	9 (45%)	21 (64%)
Throughout the year	16 (80%)*	14 (42%)
When moving in a new home	6 (30%)	8 (24%)
With another pet	5 (25%)	9 (27%)
Before a long trip	5 (25%)	8 (24%)
When there are guests	3 (15%)	3 (9%)
Other	0 (0%)	2 (6%)

Number (%) of owners selecting each situation. *p < 0.05 between groups.

### 3.4 Characteristics of diffusers

On day 7, 57% of owners using Zenifel reported their cats did not pay attention to the diffuser while 33% reported a slight interest, 9% reported a moderate interest and 2% reported a high interest (like rubbing, sniffing, turning around). By the end of the study, 78% of owners reported no interest, 15% a slight interest and 7% a moderate interest. With the reference product, 86 to 80% reported no interest between day 7 and day 60, 18 to 11% reported a slight interest and 2% reported a moderate interest.

Over the 60 days of the study, 33 and 32% of owners reported a change of odor with Zenifel and the reference product, respectively.

The products were considered easy to use by most or all owners (98% with Zenifel and 100% with the reference product).

## 4 Discussion

The aim of our study was to evaluate the efficacy of a new, non-powered, long-lasting gel diffuser of an analog of the F3 fraction of feline facial pheromone, in reducing signs of stress and stress-related problem behavior in cats, and to compare its efficacy with an existing powered diffuser of a pheromone complex. The results showed that both types of diffusers were able to significantly improve undesirable behaviors and in a similar way.

To be included in the study, first, cats had to be reported by their owners as showing at least one sign of negative emotions (fear, anxiety, suspiciousness), stress, or destructiveness such as scratching. Second, cats had to present with at least one behavior from a list of 25 commonly recognized signs of nonspecific anxiety/distress in cats, which was made based on the list assembled by Hammerle et al. (10). This list included physical indicators of stress such as mydriasis, panting, trembling and drooling; behavioral indicators of stress such as freezing and lip-licking; coping strategies such as hiding and seeking the owner’s attention; and problem behaviors associated with stress such as clawing and inappropriate elimination. This meant that we would be evaluating a very broad range of stress indicators. However, only a subset of these indicators was observed in the study population at a frequency that made them suitable for analysis. The final shorter list of specified behaviors included one problem behavior (destructive scratching), three behavioral indicators of stress (hypervigilance, repetitive meowing and inactivity) and two coping strategies (hiding and seeking the owner’s attention). This offers a more comprehensive set of observed behaviors than merely evaluating a problem behavior like destructive scratching, and provides an insight into the effects of the tested products on underlying stress. It also enables us to anticipate positive effects of the tested pheromone products on stress-related problem behaviors other than scratching.

In cats, the main functions of scratching are self-maintenance (e.g., claw sharpening and muscle stretching) and communication (e.g., visual and scent marking of territorial boundaries). As with indoor urine marking, inappropriate indoor scratch-marking can be a response to stress (19). It can become a destructive and costly nuisance when it involves household furnishings and decorations. Pheromone products that are based on the F3 fraction of facial pheromone have been used to treat a variety of behavior problems in cats, including house-soiling, aggression and scratching. For example, in a blinded, placebo-controlled study, scratching was significantly reduced in frequency and intensity by day 28, compared with baseline and control, in cats treated with an F3 facial pheromone analog diffuser (Feliway® Classic) (16).

In the first study presenting the pheromone complex diffuser (Feliway^®^ Optimum), the recruited cats had a range of problems including inappropriate urination, fear, inter-cat conflict and scratching. The frequency and intensity of these problems were significantly improved by day 28, but the study did not include a control group (20). A subsequent study tested this diffuser in a large population of 384 households (494 cats), and included a no-product control group (18). The study found improvements in scores for scratching, urine marking, fear-related issues, and cohabitation issues compared to baseline and the no-product control condition. There were also substantial reductions in the percentage of caregivers reporting the various problem behaviors at the end of the study (day 42).

The results of our study are consistent with these previous findings. We found a significant reduction over time in the percentage of participants reporting scratching for both diffuser devices (see Figure 1). In addition to scratching, our study also included an evaluation of several signs of stress. Stress is considered to be one of the major factors causing behavior problems in cats, and can significantly impact the welfare and quality of life of cats, contributing to a range of health issues (6, 7). Alleviation of stress would be expected to have a general effect on stress-related behaviors including scratching, spray-marking and elimination problems.

Synthetic pheromone analogs based on the F3 fraction of facial pheromone have been found to have positive effects on biological correlates of stress, including salivary cortisol (21), and signs of the reactivation of feline herpesvirus-1 infection in kittens (22). There is also evidence that pheromones can alleviate the signs and effects of situational stress. For example, stress vocalizations by cats, but not blood pressure, were significantly reduced in the waiting room of a veterinary clinic when a towel sprayed with an analog of F3 fraction of facial pheromone was placed over the cat carrier (23). Signs of stress were also reduced during the manipulation of cats when this type of pheromone was sprayed in the study room, with more efficiency when *Nepeta cataria* was included with the pheromone (24). In a placebo-controlled test of the effects of a feline facial pheromone product on transport stress, cats showed reduced stress signs including freezing, meowing, hiding and trembling, compared with placebo (17). Argüelles et al. found that a low-stress transport protocol, including preparing the travel basket with a F3 facial pheromone analog spray, was associated with reduced time to sedation and lower propofol dose for induction compared with a control group (25).

With both of the diffuser products tested in our study, we observed significant reductions in stress signs. The trajectories of adjusted global scores for both diffusers were visually very similar, and scores reached significance from day 7 for both products. For Zenifel^®^, a notable decrease in all six specific unwanted behaviors was observed, with significant improvements seen as early as day 7 for behaviors such as hypervigilance/hyperalertness and hiding. With the electric diffuser of pheromone complex, significant reductions were reported in four of the six behaviors monitored, including hypervigilance/hyperalertness and hiding. The earliest significant changes were found by day 15 for hypervigilance/hyperalertness. Although Zenifel showed a slightly more rapid onset, by day 60, the comparative analysis between the Zenifel and reference groups revealed no significant difference in the change in proportion of cats exhibiting each targeted behavior. This outcome suggests that, over time, both interventions offer similar efficacy in reducing unwanted behaviors in cats. The trajectories of the decreases in scratching behavior with both diffusers in our study also matched that which was observed for Feliway^®^ Optimum diffuser in the study by McPeake et al. (18). The trajectories observed for the pheromone products in our study indicated continued improvements in behavior after 30 days, especially for hypervigilance, attention seeking, hiding and scratching. Most previous studies have been of short duration, typically around 28 days, and would not have picked this up. Our findings support the recommendation in the meta-analysis by Mills et al. (15), that studies with pheromones should be two or more months in duration, in order to improve their sensitivity to effects and to make them more comparable to trials of prescription medications. In addition to reporting stress signs and behaviors, owners were also asked to report the situations in which their cats appeared calmer during treatment with the products. Cats were perceived to be calmer across a wide range of situations, including when left alone, touched or carried, with unfamiliar people, and during changes of environment. This aligns with the generally positive effect pheromones have been found to have on the stress which underlies behavioral responses.

It is interesting that owners noted increased calmness in situations away from home, including during transport and trips to the veterinary clinic. In these situations, the cat ought to have been away from the installed diffuser product for some time, so there would be no direct exposure to pheromones at that point. There are several possible explanations. Firstly, the persistent effects of pheromone exposure after separation from the diffuser. Although it is known that the pheromones bind only temporarily to sites in the vomeronasal organ, the persistence of this binding and the associated effects on behavior are not well described. Secondly, that a reduction in allostatic load from daily stress, due to the effects of the diffuser at home, might produce an improvement in situational stress responses even out of range of the diffuser. This might be mediated by reductions in fatigue, improvements in sleep or rest, and reduced perception threat exposure in everyday situations, for example. One potential mechanism for this could be related to modifications in gene expression within brain circuits involved in the regulation of stress, which could persist for some time beyond the exposure to pheromones. Thirdly, pheromone might become bound to the surface substrate of objects, including bedding and carrying baskets, so that exposure is maintained outside the home.

However, there could also be explanations relating to owner-psychology. It is also possible that owners’ positive beliefs about the effectiveness of the products created an expectation that they would have beneficial effects even in situations where the products were not present. Also, owners might have overstated the positive effects of the pheromone products because they wanted to show that their efforts in using these products were yielding good results. Although the survey questions were intentionally phrased neutrally, these effects cannot be entirely ruled out in an owner-report survey.

Either way, it does raise the issue that owners do notice the beneficial effects of pheromones outside the home, which gives a non-powered device the advantage of portability. Almost twice the percentage of owners (80% vs. 42%, *p* < 0.05) indicated that they would use the gel diffuser device (Zenifel^®^) throughout the year compared with the powered device (Feliway^®^ Optimum). For all other situations, from moving home to decreasing undesirable behaviors, the percentage of owners saying that they would use the devices was not significantly different. Unfortunately, we did not ask participants why they would use these devices all year round, so it is not clear why Zenifel^®^ would have such an advantage when in most ways the two diffusers performed similarly.

It seems likely that the unpowered nature of Zenifel^®^ diffuser made the device easier and more convenient to locate, compared with a powered device needing an available power outlet (while avoiding multi-socket outlets and extension leads), and perhaps owners felt that it could be used more flexibly in a wider range of situations. Ease of use was identified by Grigg et al. (2) as a general advantage of pheromone products, compared with herbals and other medication, and a non-powered diffuser would seem to add to this ease of use. The extended duration (2 months instead of 1 month) could also have been seen as an economic and practical advantage since less devices are required per year. The ability to place the Zenifel^®^ device in strategic locations without worrying about proximity to power outlets might mean that owners can optimize the distribution of the pheromone in the environment where the cat spends its time. This is particularly important for cat owners who may not have accessible outlets in areas where their cats spend most of their time. This spatial flexibility could improve the effectiveness of the intervention.

There may be other deterrent factors that are common to electrical devices. With rising energy prices, many consumers are trying to cut their energy bills, so the cost of operating plug-in diffusers may also be an influential factor for some owners. Despite using relatively little energy, plug-in air fresheners, which have similar energy consumption to plug-in pheromone diffusers, have been listed in the media as being among the “vampire devices” that add hidden costs to household energy bills (26). Electric diffusers, whilst safe, do carry a small risk of overheating or electrical faults if they are not used according to the manufacturer’s instructions. For example, the manufacturers of Feliway^®^ warn that the devices can get too hot if they are used on an extension lead (CEVA USA). Some owners might perceive a non-powered device to be generally safer around pets and children, avoiding concerns about leaving an electric device running unattended.

The Zenifel^®^ diffuser product contains *Nepeta cataria* extract as an attractant, to increase exploratory sniffing and thereby exposure to the pheromones. Although *Nepeta cataria*, commonly known as “catnip,” is well known as an attractant for cats, a range of other plants have been reported to have similar properties (27). These include Tatarian honeysuckle (*Lonicera tatarica*), silver vine (*Actinidia polygama*), and valerian (*Valeriana officinalis*) (27). The common effects appear to be due to the presence of nepetalactols and structurally similar actinidine in these plants (27). The biological function of these chemicals for the plant is as insect repellants, and it has been theorized that the cat’s behavioral responses to them (attraction, sniffing, chewing, licking and rolling) have the function of transferring the chemicals to the animal’s coat, where they act to deter biting insects such as mosquitos (28, 29). In the context of pheromone products, the low-concentration effects on sniffing and attraction are the reasons for their inclusion. Catnip (*Nepeta cataria*) can have behavioral effects in addition to its attractant properties. For example, an extract of *Nepeta cataria* has been reported to have a calming effect on kittens exposed to a novel environment (30) and a diffuser of *Nepeta cataria* can reduce signs like hissing or biting attempts toward other cats, and scratching doors (31). Furthermore, adding this plant extract to a spray with feline facial pheromone has been shown to increase the pheromone efficiency during an acute stressful event (24). Consistently, in our study, although there was no significant difference between the responses to both products, more behaviors were significantly reduced with the gel diffuser containing the catnip extract (6 behaviors improved with Zenifel) than with the diffuser containing the pheromone complex alone (4 behaviors improved with Feliway Optimum, Figure 1). This effect could be linked to the presence of catnip extract in the Zenifel product.

One potential criticism of the present study is the absence of a placebo group. Since the owners in both groups were aware that they were testing a product intended to calm their cats, it is likely that they were expecting such results. This could contribute to a placebo effect. The decision between using a placebo or an active control is complicated, particularly for a study that involves owned cats that are exhibiting problem behavior which is indicative of underlying stress. This consideration is especially pertinent when the study’s interventions are designed to alleviate stress or anxiety; withholding a potentially beneficial treatment in a control group raises ethical questions. Current guidance in comparable fields, such as clinical trials of medicinal products conducted with minors, is that placebo should not be used when it means withholding effective treatment (EU 536/2014). It would have been possible to use a licensed medicinal product as an active control, but no such product has been specifically licensed for the treatment of problem scratching. Using such a product on an unlicensed basis would have raised serious ethical, legal, and welfare concerns. Given the extensive background of research into the effects of pheromone products in cats, and the recently published trial demonstrating the efficacy of the pheromone complex diffuser (Feliway^®^ Optimum), it seemed appropriate to use that product as an active control.

## 5 Conclusion

This study assessed the efficacy of two different diffusers to reduce undesirable behaviors: one was a non-powered, long-lasting (2 months) gel diffusing an analog of the F3 fraction of facial pheromone, with *Nepeta cataria* (Zenifel gel diffuser) while the other was a plug-in diffuser of a pheromone complex lasting 1 month (Feliway Optimum). The results showed that both diffusers could reduce undesirable behaviors in a similar way. However, the non-powered diffuser brought some advantages that led more cat owners to believe that it could be used regularly throughout the year. This study then confirms the role of pheromone in the management of undesirable behaviors and provides evidence that a non-powered long-lasting device may be more appreciated by cat caregivers.

## Data Availability

The raw data supporting the conclusions of this article will be made available by the authors, without undue reservation.

## Footnotes

^1^ www.real-statistics.com

## References

[1] O’Neill DG Church DB McGreevy PD Thomson PC Brodbelt DC. Prevalence of disorders recorded in cats attending primary-care veterinary practices in England. Vet J. (2014) 202:286–91. doi: 10.1016/j.tvjl.2014.08.004, PMID: 25178688

[2] Grigg EK Kogan LR van Haaften K Kolus C. Cat owners’ perceptions of psychoactive medications, supplements and pheromones for the treatment of feline behavior problems. J Feline Med Surg. (2019) 21:902–9. doi: 10.1177/1098612X18807783, PMID: 30382770 PMC11132244

[3] Möstl K Egberink H Addie D Frymus T Boucraut-Baralon C Truyen U. Prevention of infectious diseases in cat shelters: ABCD guidelines. J Feline Med Surg. (2013) 15:546–54. doi: 10.1177/1098612X13489210, PMID: 23813812 PMC11148948

[4] Tanaka A Wagner DC Kass PH Hurley KF. Associations among weight loss, stress, and upper respiratory tract infection in shelter cats. J Am Vet Med Assoc. (2012) 240:570–6. doi: 10.2460/javma.240.5.570 22332626

[5] Titeux E Gilbert C Briand A Cochet-Faivre N. From feline idiopathic ulcerative dermatitis to feline behavioral ulcerative dermatitis: grooming repetitive behaviors indicators of poor welfare in cats. Front Vet Sci. (2018) 5:81. doi: 10.3389/fvets.2018.00081, PMID: 29713639 PMC5911546

[6] Amat M Camps T Manteca X. Stress in owned cats: behavioural changes and welfare implications. J Feline Med Surg. (2016) 18:577–86. doi: 10.1177/1098612X15590867 26101238 PMC10816390

[7] Buffington CAT Bain M. Stress and feline health. Vet Clin North Am Small Anim Pract. (2020) 50:653–62. doi: 10.1016/j.cvsm.2020.03.001, PMID: 32354488 PMC8801065

[8] Casey RA Vandenbussche S Bradshaw JWS Roberts MA. Reasons for relinquishment and return of domestic cats (*Felis Silvestris* Catus) to rescue shelters in the UK. Anthrozoös. (2009) 22:347–58. doi: 10.2752/089279309X12538695316185

[9] Houser B Vitale KR. Increasing shelter cat welfare through enrichment: a review. Appl Anim Behav Sci. (2022) 248:105585. doi: 10.1016/j.applanim.2022.105585

[10] Hammerle M Horst C Levine E Overall K Radosta L Rafter-Ritchie M. 2015 AAHA canine and feline behavior management guidelines. J Am Anim Hosp Assoc. (2015) 51:205–21. doi: 10.5326/JAAHA-MS-6527 26191821

[11] Vitale KR. Tools for managing feline problem behaviors: pheromone therapy. J Feline Med Surg. (2018) 20:1024–32. doi: 10.1177/1098612X18806759, PMID: 30375946 PMC11343345

[12] Torres MV Ortiz-Leal I Sanchez-Quinteiro P. Pheromone sensing in mammals: a review of the vomeronasal system. Anatomia. (2023) 2:346–413. doi: 10.3390/anatomia2040031

[13] Feliway. 25 Years of FELIWAY! Share your story with us. Feliway UK. Available at: https://www.feliway.co.uk/blogs/news/25-years-of-feliway-share-your-story-with-us-to-win-2500 (Accessed July 10, 2024)

[14] Pereira JS Salgirli Demirbas Y Meppiel L Endersby S da Graça PG De Jaeger X. Efficacy of the Feliway® classic diffuser in reducing undesirable scratching in cats: a randomised, triple-blind, placebo-controlled study. PLoS One. (2023) 18:e0292188. doi: 10.1371/journal.pone.0292188 37851638 PMC10584138

[15] Mills DS Mills CB. Evaluation of a novel method for delivering a synthetic analogue of feline facial pheromone to control urine spraying by cats. Vet Rec. (2001) 149:197–9. doi: 10.1136/vr.149.7.197, PMID: 11548956

[16] Pereira JS Fragoso S Beck A Lavigne S Varejão AS da Graça PG. Improving the feline veterinary consultation: the usefulness of Feliway spray in reducing cats’ stress. J Feline Med Surg. (2016) 18:959–64. doi: 10.1177/1098612X15599420, PMID: 26282847 PMC11112237

[17] Shu H Gu X. Effect of a synthetic feline facial pheromone product on stress during transport in domestic cats: a randomised controlled pilot study. J Feline Med Surg. (2022) 24:691–9. doi: 10.1177/1098612X211041305, PMID: 34493099 PMC10812279

[18] McPeake K Sparkes A Billy C Endersby S Collin JF De Jaeger X. Development of a cat behaviour issues assessment scale (CABIAS) assessing problem behaviours in cats. Animals. (2023) 13:2992. doi: 10.3390/ani13182992, PMID: 37760392 PMC10525805

[19] DePorter TL Elzerman AL. Common feline problem behaviors: destructive scratching. J Feline Med Surg. (2019) 21:235–43. doi: 10.1177/1098612X19831205, PMID: 30810089 PMC11373750

[20] De Jaeger X Meppiel L Endersby S Sparkes AH. An initial open-label study of a novel pheromone complex for use in cats. Open J Vet Med. (2021) 11:105–16. doi: 10.4236/ojvm.2020.113006

[21] da Silva BPL Knackfuss FB Labarthe N Mendes-de-Almeida F. Effect of a synthetic analogue of the feline facial pheromone on salivary cortisol levels in the domestic cat. Pesqui Vet Bras. (2017) 37:287–90. doi: 10.1590/s0100-736x2017000300013

[22] Contreras ET Hodgkins E Tynes V Beck A Olea-Popelka F Lappin MR. Effect of a pheromone on stress-associated reactivation of feline Herpesvirus-1 in experimentally inoculated kittens. J Vet Intern Med. (2018) 32:406–17. doi: 10.1111/jvim.14894, PMID: 29219213 PMC5787191

[23] Van Vertloo LR Carnevale JM Parsons RL Rosburg M Millman ST. Effects of waiting room and feline facial pheromone experience on blood pressure in cats. Front Vet Sci. (2021) 8:640751. doi: 10.3389/fvets.2021.640751 33748216 PMC7973014

[24] Bernachon N Beata C Crastes N Monginoux P Gatto H McGahie D. Response to acute stress in domestic cats using synthetic analogues of natural appeasing pheromones with *Nepeta cataria* extract rich in nepetalactone: a double-blinded, randomized, positive controlled cross-over study. Int J Appl Res Vet Med. (2015) 13:125–34.

[25] Argüelles J Echaniz M Bowen J Fatjó J. The impact of a stress-reducing protocol on the quality of pre-anaesthesia in cats. Vet Rec. (2021) 188:e138. doi: 10.1002/vetr.138, PMID: 33645705

[26] evie-townend. “Vampire devices” that are adding £189 to your energy bills each year – how to cut costs. The Mirror. (2022). Available at: https://www.mirror.co.uk/money/vampire-devices-adding-189-your-27753072 (Accessed June 24, 2024).

[27] Bol S Scaffidi A Bunnik EM Flematti GR. Behavioral differences among domestic cats in the response to cat-attracting plants and their volatile compounds reveal a potential distinct mechanism of action for actinidine. BMC Biol. (2022) 20:192. doi: 10.1186/s12915-022-01369-1, PMID: 36008824 PMC9414117

[28] Uenoyama R Miyazaki T Hurst JL Beynon RJ Adachi M Murooka T. The characteristic response of domestic cats to plant iridoids allows them to gain chemical defense against mosquitoes. Sci Adv. (2021) 7:eabd9135. doi: 10.1126/sciadv.abd9135, PMID: 33523929 PMC7817105

[29] Uenoyama R Miyazaki T Adachi M Nishikawa T Hurst JL Miyazaki M. Domestic cat damage to plant leaves containing iridoids enhances chemical repellency to pests. iScience. (2022) 25:104455. doi: 10.1016/j.isci.2022.104455, PMID: 35880027 PMC9308154

[30] Marchei P Diverio S Falocci N Fatjó J Ruiz-de-la-Torre JL Manteca X. The effect of *Nepeta cataria* on kittens’ behavior. J Vet Behav. (2010) 5:50–1. doi: 10.1016/j.jveb.2009.09.022

[31] Cannas S Scaglia E Talamonti Z Dallara P Palestrini C. Effect of a *nepeta cataria* oil diffusor on cat behaviour. Veterinaria. (2018) 32:43–9.

