# Supplementary material for: A long-lasting gel-based diffuser of feline pheromone can help reduce undesirable behaviors in cats at home: comparison with an electric diffuser
Source: Front Vet Sci. 2024 Aug 29;11:1445108. doi: 10.3389/fvets.2024.1445108 (PMC11390375; doi:10.3389/fvets.2024.1445108)
Supplement: Supplementary file 1 [file Table_1.DOCX]

Supplementary Material

**Supplementary Table 1.** Percentage of owners reporting a specific behavior. * p<0.05 (Fisher’s exact test).

| **Behavior** | **Zenifel gel diffuser**  (n=46) | **Reference electric diffuser** (n=44) |
| --- | --- | --- |
| Hypervigilance/hyperalertness | 54% | 66% |
| Seeking owner’s attention | 41% | 48% |
| Hiding or trying to hide | 35% | 34% |
| Repetitive or excessive meowing out of context | 30% | 27% |
| Immobility, few activities | 28% | 18% |
| Excessive scratching | 20% | 18% |
| Raises its hair, contracts skin | 7% | 20% |
| Repetitive activities (turn in circle, catch tail, go forth and back…) | 9% | 18% |
| Tries to escape | 7% | 18% |
| Hyperactive | 9% | 11% |
| Overgrooming | 2% | 14% |
| Dilated pupils (mydriasis) | 9% | 7% |
| Urinates in an unusual/inappropriate place | 4% | 9% |
| Decreases contact with owner | 9% | 5% |
| Often licks lips and nose | 2% | 9% |
| Defecates in an unusual/inappropriate place | 2% | 9% |
| Smacks/pops lips or jaws together | 0% | 11%* |
| Does not tolerate direct eye contact | 4% | 5% |
| Retracts lips/grimaces | 2% | 5% |
| Decreased grooming | 2% | 5% |
| Carries the head and/or the neck low, tail between the legs | 2% | 5% |
| Panting (even if not hot weather) | 0% | 5% |
| Often discharges anal glands | 2% | 0% |
| Trembles a lot | 2% | 0% |
| Drools excessively (hypersalivation) | 0% | 0% |

**Supplementary Table 2**: Situations where unwanted behaviors are observed

| **Situation** | **Zenifel gel diffuser**  (n=46) | **Reference electric diffuser** (n=44) |
| --- | --- | --- |
| Loud noise (firework, thunder, hoover…) | 61% | 70% |
| With unfamiliar people | 57% | 52% |
| During a visit to the veterinarian or other person | 41% | 25% |
| When feeling threatened | 37% | 43% |
| Change of enviornment | 33% | 27% |
| During transport (car or other) | 26% | 36% |
| When forced to stay in a particular place or cage | 22% | 25% |
| No particular reason | 22% | 20% |
| When left alone | 20% | 18% |
| With other animals | 17% | 32% |
| When touched or carried | 13% | 20% |
| Other | 54% | 61% |
